# Mapping the Landscape: Simulation Centers in Portugal

**DOI:** 10.7759/cureus.56278

**Published:** 2024-03-16

**Authors:** Bruno Miguel Silva, Gustavo Norte, Pedro Lito, Pedro Garcia

**Affiliations:** 1 Medical Oncology Department, Hospital de Loures, Unidade Local de Saúde de Loures-Odivelas, Loures, PRT; 2 Pathophysiology Department, NOVA Medical School, Universidade NOVA de Lisboa, Lisboa, PRT; 3 Anesthesiology Department, Unidade Local de Saúde de Trás-os-Montes e Alto Douro, Vila Real, PRT; 4 Critical Care Unit, Unidade Local de Saúde da Cova da Beira, Covilhã, PRT; 5 Medical Sciences Department, Universidade da Beira Interior, Covilhã, PRT; 6 Neonatal Intensive Care Unit, Hospital de Dona Estefânia, Unidade Local de Saúde São José, Lisboa, PRT; 7 Pediatrics Department, NOVA Medical School, Universidade NOVA de Lisboa, Lisboa, PRT; 8 Simulation Center, CUF Academic Center, Lisboa, PRT

**Keywords:** simulation-based-education, simulation research, education and training, national policy, healthcare simulation

## Abstract

Introduction: Simulation-based training has emerged as a vital component of healthcare education. This study aims to characterize Portuguese simulation centers concerning their geographic distribution and key features, providing stakeholders with valuable insights to inform strategic decisions.

Methods: A cross-sectional survey-based study was conducted over two years (2021-2023) to investigate the geographical dispersion and characteristics of simulation centers in Portugal. Descriptive statistics and thematic analysis were used to analyze data.

Results: Twenty-three Portuguese simulation centers were included. Major urban areas and coastal regions bring together 20 simulation centers (86.96%). A large percentage (71.93%) of centers were affiliated with academic institutions, while five centers (21.74%) were clinically affiliated. Emergency care, Anesthesiology and Intensive Medicine, Pediatrics, and Gynecology and Obstetrics were identified as the national key areas of intervention.

Discussion: Significant geographical disparity raises concerns about unequal access to professional training opportunities using simulation. Centers should be encouraged to incorporate developing technologies and innovative pedagogical methodologies and to expand their training repertoire into relatively uncharted territories.

Conclusion: Several issues have been identified within the national simulation network. Stakeholders and policymakers should prioritize equitable access, bolster the prevalence of clinical affiliated centers, foster innovation, and facilitate strategic coordination.

## Introduction

Simulation-based training has emerged as an essential resource for enhancing healthcare education and improving patient outcomes. Simulation offers healthcare professionals practical and controlled environments where they can perform challenging medical procedures, perfect decision-making skills, and establish successful teamwork [1]. In simulation settings, training by repetition is feasible and independent of the case-mix, and feedback can be promptly provided in a safe environment [2]. There is already extensive research demonstrating the value of simulation in pre- and post-graduate health sciences education, both for technical and non-technical skill development [3].

In Portugal, the landscape of medical simulation is continuously evolving, with a growing interest in leveraging simulation technology to enhance the competency and confidence of healthcare professionals [4]. Remarkably, there are over 20 simulation centers in Portugal, serving military, academic, and clinical needs, comprising both publicly and privately held facilities. Understanding the current geographical distribution and characteristics of the Portuguese simulation centers has significant implications for healthcare and medical education stakeholders, such as policymakers, medical educators, and healthcare administrators. This analysis could highlight regional disparities in access to simulation training and help to identify potential areas for improvement.

We present a cross-sectional study that aims to provide an overview of the current landscape of Portuguese simulation centers. The specific objectives of this study include analyzing the geographic distribution of simulation centers as well as providing an overall characterization of their activities and educational offerings, with a focus on identifying potential areas for improvement.

## Materials and methods

Study design

A cross-sectional study design was employed to examine the geographical distribution and characteristics of simulation centers in Portugal. The study was conducted over a period of two years, from August 2021 to August 2023.

Survey development

The questionnaire was developed using a modified Delphi panel method to ensure the inclusion of relevant information pertinent to the study objectives. Four clinicians with experience in simulation-based teaching (between five and 20 years of experience) were involved in designing the questionnaire. The process involved four rounds of adjustments until a consensus was reached. Additionally, the questionnaire underwent informal pilot testing to assess clarity and comprehensiveness.

Sampling methodology

The study required the completion of a questionnaire by coordinators of Portuguese simulation centers. All Portuguese organizations equipped with dedicated space, staff, and resources tailored for simulation interventions within the Health Sciences domain were considered eligible. A convenience sample was obtained by distributing online self-report questionnaires through various channels, including the official website of the Portuguese Society for Simulation in Health Sciences (SPSim), personalized emails to simulation center coordinators whose contacts were obtained through research on institutional websites, social media platforms, and announcements during the national simulation conference. A ​​snowball sampling technique was also used to ensure the participation of centers whose institutional contact was not known. Simulation centers that participated in the study were promised visibility by having their information published on the SPSim website, aimed at incentivizing participation.

Data collection

Data were collected through an online self-report survey that gathered information about simulation centers, including contact details, scope, objectives, facility features, coordinator information, target training levels, available technologies, and subject areas covered in training programs. Participants' informed consent was obtained before data submission. ​​Throughout the study period, centers were allowed to revise the previously submitted data to keep the record as up-to-date as possible. The database was locked on August 31, 2023.

Data management and analysis

Data collected from the survey were processed and analyzed using Microsoft Excel® (version 16.82; Microsoft Corp., Armonk, NY, USA) for initial data management and IBM SPSS Statistics® (version 29; IBM Corp., Redmond, Washington, USA) for detailed statistical analysis. Descriptive statistics were utilized to analyze quantitative data, while qualitative data obtained from open-ended survey questions underwent inductive thematic analysis.

Geographic mapping

Simulation centers were categorized by districts and autonomous regions of Portugal. For graphic representation, a vector map of Portugal sourced from https://freevectormaps.com was utilized and modified using Photoshop (version 25.4) [5]. The map was color-coded to represent the density of simulation centers, with a graduated scale used to depict variations in density. The geographical positions of the autonomous regions of Madeira and Azores were adjusted to condense information within a smaller space while maintaining visual clarity.

## Results

Geographic distribution

Throughout the study's duration, 23 Portuguese simulation centers were included. Among these, nine centers (39.13%) were located in the Lisbon and Tagus Valley region, six centers (26.09%) in the Center region, five centers (21.74%) in the North, two centers (8.70%) in the Autonomous Region of Madeira, and one center (4.35%) in the Algarve.

**Figure 1:**
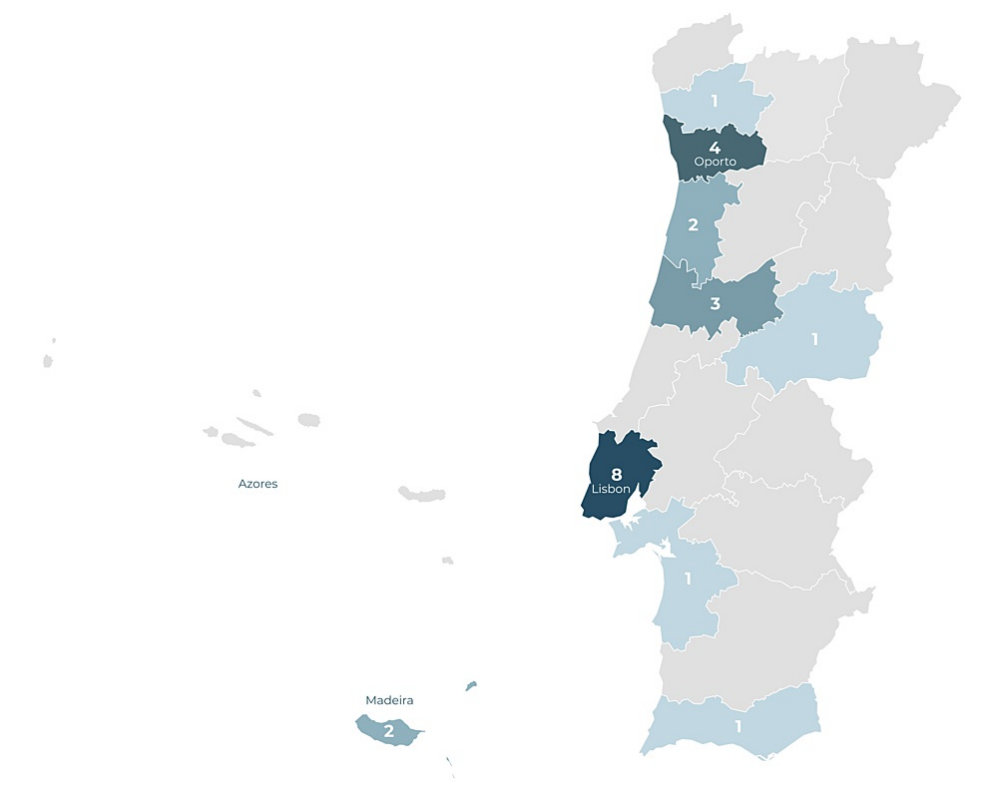
Distribution of simulation centers throughout Portugal Darker tones indicate a higher density of simulation centers. The dominance of major urban centers (Lisbon and Oporto) and coastline areas is worth noting, as opposed to the obvious scarcity in the country's most inland region. (Map vector source: https://freevectormaps.com [5].)

The major metropolitan areas, namely Lisbon and Oporto, collectively accounted for 14 simulation centers (60.87%). Remarkably, only one center (4.35%) was located inland, while the remaining 20 centers (86.96%) were positioned along the coastal districts. Two simulation centers were located on Madeira Island, and no simulation centers were reported in the Azores territory. A graphical representation of the distribution of simulation centers across various districts and autonomous regions in Portugal is provided in Figure 1.

Affiliation and coordination

Among the featured simulation centers in this study, a majority of 17 centers (71.93%) were affiliated with academic institutions. Additionally, five centers (21.74%) were clinical (two private facilities and three public hospitals), and one center (4.35%) operated within a military context.

Regarding coordinator demographics, 13 centers (56.52%) were headed by male coordinators. In terms of coordinators' background training, a majority of 20 (86.96%) had a medical or nursing background, while only one coordinator (4.35%) had a management background.

Scope and main activities

Teaching and training emerged as the central focus for all 23 (100%) simulation centers. Additionally, 19 centers (82.61%) indicated their engagement in research activities, while seven centers (30.43%) reported involvement in technology development. Furthermore, one center (4.35%) specified its role in testing new equipment and clinical protocols. Detailed data are summarized in Table 1.

**Table 1:** Characteristics of Portuguese simulation centers

Characteristics	
Center’s affiliation	No. (%)
Academic	17 (71.93)
Clinical	5 (21.74)
Military	1 (4.35)
Center’s nature	No. (%)
Public	21 (13.04)
Private	2 (8.70)
Coordinators’ demographics	No. (%)
Male	13 (56.52)
Female	10 (43.48)
Coordinators' background training	No. (%)
Medical doctor	17 (71.93)
Nurse	3 (13.04)
Biomedical engineer	1 (4.35)
Manager	1 (4.35)
Physiotherapist	1 (4.35)
Main activities	No. (%)
Education and training	23 (100.00)
Research	19 (82.61)
Simulation technology development	7 (30.43)
Other	1 (4.35)
Educational levels	No. (%)
Post-graduate	22 (95.65)
Continuing education	22 (95.65)
Pre-graduate	19 (82.61)
Target groups	No. (%)
Medical doctors and medical students	22 (95.65)
Nurses and nursing students	22 (95.65)
Technicians	19 (82.61)
Faculty	18 (78.26)
Other professionals	15 (65.22)
Pharmacists	12 (52.17)
Psychologists	10 (43.48)
Biomedical engineers	8 (34.78)

Simulation modalities

**Figure 2:**
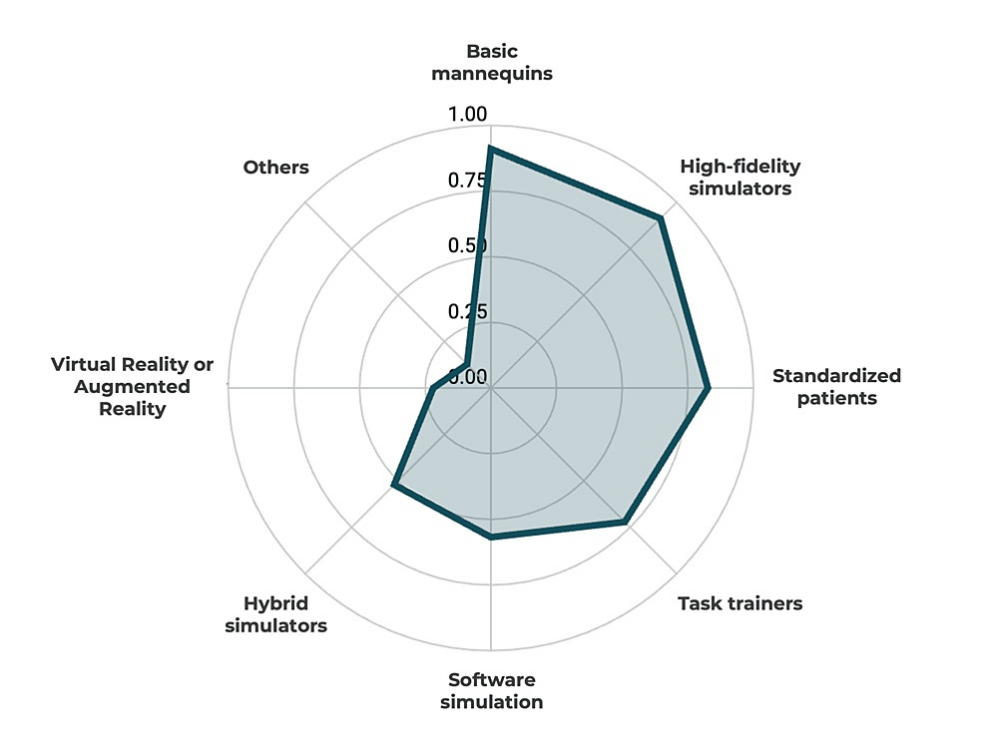
Simulation modalities

The most reported simulation modalities were the use of basic mannequins (91.30%), high-fidelity simulators (91.30%), and standardized patients (82.61%). Figure 2 illustrates the predominant simulation modalities currently used in Portugal.

Educational offer

Concerning the educational levels offered, 18 centers (78.26%) provided training across pre-graduate, post-graduate, and continuing education domains. Noteworthy, three centers (13.04%) exclusively offered post-graduate training and continuing education, while one center (4.35%) focused solely on continuing education. The majority of centers (95.65%) prioritize training doctors, nurses, and pre-graduate students.

Thematic analysis of the key areas of the intervention report revealed that the most prevalent themes are Emergency care (including pre-hospital care), Anesthesiology and Intensive Medicine, Pediatrics, and Gynecology and Obstetrics. The main themes identified and their respective prevalence are shown in Table 2.

**Table 2:** Summary of thematic analysis findings on key intervention areas

Educational Offer	No. (%)
Emergency care (including pre-hospital care)	16 (69.57)
Anesthesiology or Intensive Medicine	14 (60.87)
Pediatrics	12 (52.17)
Gynecology and Obstetrics	12 (52.17)
Other areas of Health (Pharmacy, Psychology, or Technicians)	10 (43.48)
Surgical specialties	9 (39.13)
Nursing	8 (34.78)
Other medical specialties (Neurology, Gastroenterology, Psychiatry, Cardiology, Pulmonology, Internal Medicine, or Oncology)	8 (34.78)
General and Family Medicine	7 (30.43)
Non-technical skills	7 (30.43)
Ultrasound	5 (21.74)
Medical semiology and basic procedures training	4 (17.39)
Military Health	1 (4.35)

## Discussion

This study's findings shed light on significant aspects of Portuguese simulation centers, offering insights into their distribution, affiliations, scope, and activities. The observed geographic distribution of Portuguese simulation facilities is characterized by clustering in major urban centers and coastal regions. This geographical pattern is in alignment with the disparities in regional development and several economic, social, and demographic indicators and potentially leads to unequal access to professional training opportunities for healthcare professionals [6]. This significant geographical disparity raises concerns about healthcare professionals in inland regions being potentially disadvantaged. Addressing this imbalance is imperative for promoting inclusive and widespread access to state-of-the-art medical education. Policy interventions may be necessary to encourage the establishment of centers in underserved areas, ensuring that high-quality training is accessible to all practitioners, regardless of their geographic location. Strategic partnerships between clinical centers in underserved areas and existing simulation centers may also be a strategy to explore.

The prominence of academic affiliations underscores the exemplary integration of simulation within medical educational frameworks. In this regard, it is noteworthy that all Portuguese medical schools currently have a simulation center. On the other hand, we believe the number of national nursing schools that use simulation for educational purposes is underestimated in this study. This phenomenon may be because there are no formally established simulation centers in these schools, with simulation being used on a punctual basis, or due to a bias related to the participation of nursing schools in this study. Additional studies are needed to clarify this issue. It is also critical to work on multiprofessionality within simulation facilities and partnerships between medical schools, nursing schools, and other allied healthcare schools [7-8].

The scarcity of clinical simulation centers is also a compelling opportunity for improvement. The development of dedicated simulation centers within healthcare facilities could facilitate specialized and contextualized training, enabling healthcare teams to refine their skills in environments mirroring real clinical settings. This study emphasizes the need for further expansion in this direction. Hospitals, as key providers of patient care, have the potential to amplify the impact of simulation-based training by integrating it into their daily operations. The concept of "in situ" simulation, where training scenarios are conducted within actual clinical spaces, also holds promise in enhancing team dynamics, identifying latent safety threats, and refining responses to critical situations [9]. Furthermore, the concept of "translational simulation" involves a direct improvement of patient care and healthcare systems, through analyzing safety and performance issues and providing simulation-based interventions [10]. These strategies can bridge the gap between simulated learning and real-world practice, thus fortifying healthcare teams' preparedness and fostering a culture of continuous improvement in patient care.

Regarding the centers’ educational offers, the remarkable distribution across all educational levels attests to simulation versatility. However, a tangible opportunity lies in expanding the training repertoire into relatively uncharted clinical territories. This expansion could also extend beyond healthcare professionals to encompass the general public, improving health literacy and encouraging overall well-being [11].

To retain relevance and efficacy, it is also paramount for simulation centers to transform into innovation incubators. In this study, we observed a clear commitment to the educational aspect at the expense of this point of view. Incorporating developing technologies and innovative pedagogical methodologies elevates the caliber of education delivered. Simulation centers can greatly contribute to breakthroughs in healthcare education by establishing an environment that encourages experimentation and continuous improvement, as well as fostering cross-disciplinary collaboration among simulation experts, technological pioneers, and healthcare practitioners.

In reviewing the existing literature, our study contributes significantly by addressing a notable gap, as few studies have undertaken an examination of regional simulation center networks [12-14]. A notable precedent was set by the largest study in this domain, encompassing 149 simulation centers across Latin American countries [12]. Similar to our findings, most centers (84%) were academically affiliated, with a limited representation of clinical affiliations. The study also revealed a predominant management by health professionals, mainly doctors and nurses, and a common reliance on high-fidelity simulators and task trainers (81% and 79%, respectively). Furthermore, insights from a study involving 15 simulation centers in Beijing echoed our findings, emphasizing the need for greater variability in training offerings [13]. Another international study reflects conclusions similar to ours regarding the most used simulation modalities and the need to invest in research activities [14]. The participation of nursing schools in this study was more significant.

While our study provides valuable insights, acknowledging its limitations is also crucial for a comprehensive understanding of the implications of the findings. One potential limitation lies in the possibility of response bias among non-responding simulation centers. Furthermore, relying on self-reporting for data collection introduces the potential for inaccuracies due to recall biases or subjective interpretations. Additionally, the presence of missing data can impact the completeness of the analysis. Certain variables might be underrepresented due to incomplete responses, potentially affecting the conclusions' robustness. To mitigate these limitations in future research, complementary methodologies could be considered, such as on-site visits and external audits, to enhance the credibility and validity of the findings. Employing quality criteria to develop the survey, such as those utilized in the study conducted by Armijo-Rivera et al. [12], represents a valuable strategy for standardizing information that should be considered in future analyses. This approach enhances the reliability of data collection methods and facilitates meaningful comparisons between studies within the field.

Considering the cross-sectional design used, follow-up studies are needed to track changes over time, capturing trends, adaptations, and advancements in simulation-based education. A network analysis should also be considered since it can investigate the relationships between simulation centers, academic institutions, clinical settings, and other relevant partners, shedding light on how centers are interconnected and how information flows throughout the simulation community [15].

## Conclusions

This study provides crucial insights into Portugal's simulation centers, highlighting geographic disparities. To ensure comprehensive healthcare education, policymakers must prioritize equitable access, bolster clinical affiliations, foster innovation, and facilitate strategic coordination. By committing to these principles, stakeholders can ensure a well-rounded healthcare education, seamless integration of skills, adoption of cutting-edge learning methods, and a cohesive approach to healthcare education and practice in Portugal.
